# Drivers’ Mental Engagement Analysis Using Multi-Sensor Fusion Approaches Based on Deep Convolutional Neural Networks

**DOI:** 10.3390/s23177346

**Published:** 2023-08-23

**Authors:** Taraneh Aminosharieh Najafi, Antonio Affanni, Roberto Rinaldo, Pamela Zontone

**Affiliations:** Polytechnic Department of Engineering and Architecture, University of Udine, Via Delle Scienze 206, 33100 Udine, Italy; antonio.affanni@uniud.it (A.A.); roberto.rinaldo@uniud.it (R.R.); pamela.zontone@uniud.it (P.Z.)

**Keywords:** sensor fusion, drivers’ mental engagement, electroencephalogram, electrodermal activity, electrocardiogram, deep convolutional neural network

## Abstract

In this paper, we present a comprehensive assessment of individuals’ mental engagement states during manual and autonomous driving scenarios using a driving simulator. Our study employed two sensor fusion approaches, combining the data and features of multimodal signals. Participants in our experiment were equipped with Electroencephalogram (EEG), Skin Potential Response (SPR), and Electrocardiogram (ECG) sensors, allowing us to collect their corresponding physiological signals. To facilitate the real-time recording and synchronization of these signals, we developed a custom-designed Graphical User Interface (GUI). The recorded signals were pre-processed to eliminate noise and artifacts. Subsequently, the cleaned data were segmented into 3 s windows and labeled according to the drivers’ high or low mental engagement states during manual and autonomous driving. To implement sensor fusion approaches, we utilized two different architectures based on deep Convolutional Neural Networks (ConvNets), specifically utilizing the Braindecode Deep4 ConvNet model. The first architecture consisted of four convolutional layers followed by a dense layer. This model processed the synchronized experimental data as a 2D array input. We also proposed a novel second architecture comprising three branches of the same ConvNet model, each with four convolutional layers, followed by a concatenation layer for integrating the ConvNet branches, and finally, two dense layers. This model received the experimental data from each sensor as a separate 2D array input for each ConvNet branch. Both architectures were evaluated using a Leave-One-Subject-Out (LOSO) cross-validation approach. For both cases, we compared the results obtained when using only EEG signals with the results obtained by adding SPR and ECG signals. In particular, the second fusion approach, using all sensor signals, achieved the highest accuracy score, reaching 82.0%. This outcome demonstrates that our proposed architecture, particularly when integrating EEG, SPR, and ECG signals at the feature level, can effectively discern the mental engagement of drivers.

## 1. Introduction

According to the World Health Organization (WHO), road accidents are one of the leading causes of death worldwide, resulting in approximately 1.35 million fatalities annually. Many of these accidents could be prevented with proper measures [1]. Assessing the mental state of drivers, particularly their level of engagement in the driving tasks, can significantly contribute to accident prevention and save lives. Wearable biosensors offer the potential to estimate drivers’ mental and emotional states [2,3,4,5], providing valuable insights into their level of engagement during driving tasks [6,7]. However, measuring physiological signals in real world scenarios can present challenges, and relying on a single sensor may have limitations such as sensor failure, limited spatial or temporal coverage, imprecision, and uncertainty [8]. Sensor fusion techniques offer several advantages, including a higher Signal-to-Noise Ratio (SNR), reduced uncertainty, improved robustness, and enhanced precision [9].

Sensor fusion involves combining data [10] or information [11] from multiple sensors to achieve a more comprehensive sensing capability, enabling higher precision in tasks such as localization [12], estimation [13], recognition [14], or classification [15]. Sensors to be fused can either be homogeneous and redundant or heterogeneous and complementary. Sensor fusion can be performed at different levels. Data-level fusion, also known as early fusion, combines synchronized sensor data at the signal level. Feature-level fusion combines the most significant features of sensor data to improve decision accuracy. Decision-level fusion, or late fusion, combines the decisions of multiple sensors to achieve a more reliable and robust outcome [9].

The implementation of sensor fusion in wearable biosensors presents various requirements, including the need to minimize the number of sensors while maintaining satisfactory efficiency and ensuring optimal wearability and user comfort. Additionally, sensors must operate within a synchronized Body Sensor Network (BSN) [16], which shares similarities with Wireless Sensor Networks (WSNs) [17], but also has differences in terms of sensor placement, power consumption, and communication protocols [9]. Through the effective integration of data from wearable biosensors via sensor fusion, a comprehensive understanding of the driver’s mental state and level of engagement can be attained, leading to improved driving performance and safety on the roads.

Sensor fusion is an emerging field with widespread applications in various domains. An area that has gained significant attention from researchers is sensor fusion for the assessment of mental and emotional states. Several studies have explored this area, employing different fusion techniques and sensor modalities. For instance, the authors of [18] utilized feature and decision fusion techniques to evaluate the emotional states of subjects by incorporating multiple physiological signals such as ECG, EDA, Respiration (RSP), and blood volume pulse. Their findings indicate that the feature fusion approach yields the highest classification accuracy while maintaining a manageable computational complexity. In another study [19], researchers introduced a novel approach for feature and decision fusion of EEG and ECG signals to estimate the mental workload. Their method incorporates feature weight-driven fusion, resulting in an improved classification accuracy in terms of both feature and decision fusion. Furthermore, the mental workload of drivers was assessed in [20] using EEG, ECG, EDA, and RSP signals. These signals were integrated at the data level to classify four mental load classes. In this study, ConvNets combined with Long Short-Term Memory (LSTM) networks achieved a 97.8% accuracy using 3 s signal intervals. In yet another study [21], the mental workload level of drivers was assessed using ECG, EDA, and RSP signals. The authors found that the best classification result was obtained through a Random Forest (RF) model by fusing features extracted from EDA and RSP signals over 4 min time windows. Moreover, in [22], researchers introduced a comprehensive framework to assess driver stress by employing a feature fusion approach. The framework combined ocular indices, vehicle dynamics data, and environmental data using attention-based ConvNets and LSTM models, achieving an accuracy of 95.5%. In [23], a multimodal Deep Learning (DL) model was proposed to detect the mental states of pilots during flight simulations. This model involved the concatenation of the features extracted from ECG, RSP, and EDA signals using LSTM models. These features were then combined with the extracted features from the EEG signals using a ConvNet model. By exploiting complementary information from different sensor modalities, the proposed model achieved an accuracy of 85.2%. Lastly, the study conducted in [2] employed Electromyogram (EMG), ECG, RSP, and EDA signals to evaluate the emotional states of car racing drivers. By integrating features from all sensors using a Support Vector Machine (SVM) classifier, they achieved an accuracy of 79.3%.

The majority of the studies presented in the literature have relied on commercially available sensors to measure physiological signals from the tested subjects. However, this approach poses a limitation when it comes to accurate synchronization between different modalities. In such cases, offline synchronization between sensors from various manufacturers with different proprietary software requires manual post-processing, which can be quite challenging. To overcome this limitation, we developed our custom-designed sensors along with a customized GUI. This solution enabled us to achieve real-time synchronization with time alignment accuracy of up to 50 ms, effectively addressing the synchronization challenge.

In this study, we aimed to address the following critical questions:

(1) Can ConvNets effectively extract essential features from raw signals of multi-modal wearable biosensors for the accurate classification of drivers’ mental engagement levels?
(2) Which fusion approach, data-level or feature-level, is superior in integrating biosignals for classification?
(3) What is the impact of integrating the SPR and ECG signals with the EEG signals for each fusion approach?

To answer these questions, we developed a synchronized BSN consisting of our custom-designed EEG, SPR, and ECG sensors, all operating under the UDP protocol. To ensure efficient data collection and real-time synchronization, we designed a custom GUI. An experiment was designed to induce high and low mental engagement levels in participants during manual and autonomous driving scenarios, respectively. By utilizing the described sensor setup, we conducted an experiment involving 19 subjects and collected their biosignals. The acquired data went through motion artifact removal and pre-processing steps before being used to train and test two distinct DL architectures based on the Braindecode Deep4 ConvNet model (BD-Deep4) [24]. The first architecture employed a data-level fusion approach, treating the synchronized data as a 2D array input to the model. Additionally, we proposed a second novel architecture that utilized a feature-level fusion approach to process data from each sensor through separate branches of the same ConvNet model and then concatenate the extracted features. To evaluate the impact of sensor fusion on both architectures, we tested various sensors, starting with only six-channel EEG signals. Subsequently, we added SPR and ECG signals, and finally, we combined all three sensor signals. The performance of each configuration was assessed using LOSO cross-validation. The first architecture achieved the highest accuracy of 74.0% when combining the EEG and ECG signals, which was very close to the accuracy level of 73.0% obtained when using the combination of all sensor signals. On the other hand, the second architecture achieved the highest accuracy of 82.0% when integrating features from all sensor signals (EEG, SPR, ECG). These results suggest that our proposed architecture, which combines EEG, SPR, and ECG features, yields the most promising outcomes for classifying the mental engagement of drivers.

Our main contributions can be summarized as follows:

(1) We implemented a custom BSN for our biosensors (EEG, SPR, ECG) to collect their signals simultaneously.
(2) We developed a real-time GUI for signal collection and synchronization from all sensors, ensuring accurate time alignment of 50 ms.
(3) We conducted an extensive experiment to evaluate driver engagement levels in manual and autonomous driving scenarios using synchronized sensors.
(4) We applied pre-processing techniques to enhance the quality of sensor signals by reducing noise and artifacts.
(5) We implemented DL architectures on raw biosignals for data fusion and feature fusion using deep ConvNets. The feature fusion approach, utilizing our proposed architecture, achieved the highest accuracy, demonstrating the effectiveness of feature-level fusion for assessing driver engagement levels.
(6) We evaluated the influences of individual sensor signals and the integration of multiple sensors on the fusion performance. The best results were obtained by integrating features from all sensors (EEG, SPR, ECG) using our proposed ConvNet fusion architecture.

The paper is structured as follows: Section 2 introduces the sensors used in our experiment, the signal synchronization method, the pre-processing steps, and the employed sensor fusion architectures for the data fusion and feature fusion approaches. This section also outlines the experimental setup, which enabled us to acquire physiological signals from the drivers during simulated driving scenarios at our university. In Section 3, we present the experimental outcomes for engagement-level classification using various configurations in data fusion and feature fusion architectures. Section 4 delves into a comprehensive discussion of the results and their implications. Finally, in Section 5, we summarize the key findings and conclusions drawn from this study.

## 2. Materials and Methods

**Figure 1:**
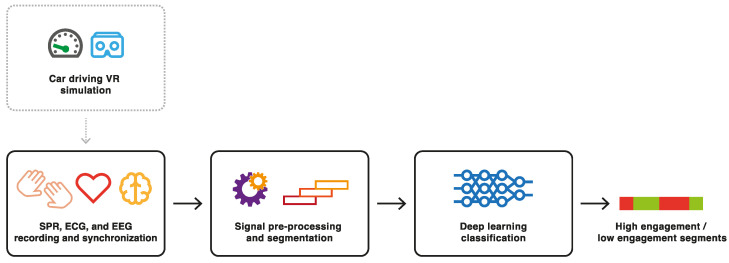
Block diagram of the implemented system.

In this section, we provide an overview of the acquisition process for the various sensors used in the experiment, the experimental setup, the sensor synchronization method, the pre-processing steps applied to the recorded physiological signals, and the sensor fusion architectures employed for signal classification. Figure 1 shows the block diagram of the proposed framework. Six EEG signals were extracted from the frontal region (Fp1, Fp2), the central region (C3, C4), and the occipital region (O1, O2) of the subject’s head. Two SPR signals were recorded from each subject’s hands. Finally, two ECG signals were recorded from each subject’s chest. These signals were collected and synchronized in real time by a custom-designed GUI. Following a pre-processing phase, the signals were used as input for two DL architectures based on data-level fusion and feature-level fusion for classification purposes. The DL models generated output labels indicating whether the subjects were mentally highly engaged or not within a specific time interval. In the following sections, each of these blocks is described in more detail, including information on the experimental setup.

### 2.1. Descriptions of the Sensors

**Figure 2:**
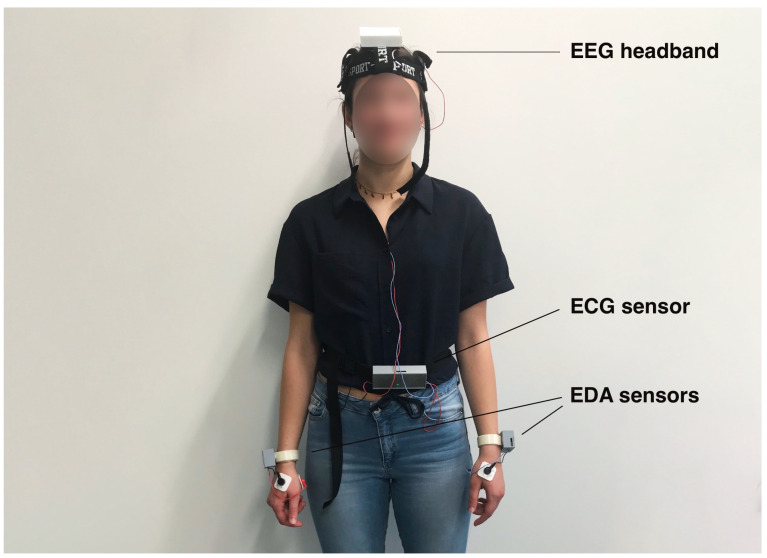
A subject wearing the EEG headband, the dual channel ECG sensor, and the two SPR sensors used in our experiment.

The proposed BSN consists of a six-channel EEG headband, two SPR sensors, and a dual-channel ECG sensor. These sensors were specifically developed by the authors and their detailed specifications and performance levels are described in [25,26]. Figure 2 shows a subject wearing the sensors, providing a visual representation of their placement and usage. The custom-designed sensors provide several advantages compared to commercially available sensors, including (1) access to raw data, (2) accurate synchronization of different signal modalities with time alignment up to 50 ms, (3) real-time integration of sensor signals facilitated by a custom-designed GUI, and (4) full sensor characterization, providing resolutions of 180 nV, 5 μV, and 2 μV, and linearities of 0.4% (6 μV), 0.15% (30 μV), and 0.05% (5 μV) for the EEG, SPR, and ECG sensors, respectively.

#### 2.1.1. EEG Sensor

**Figure 3:**
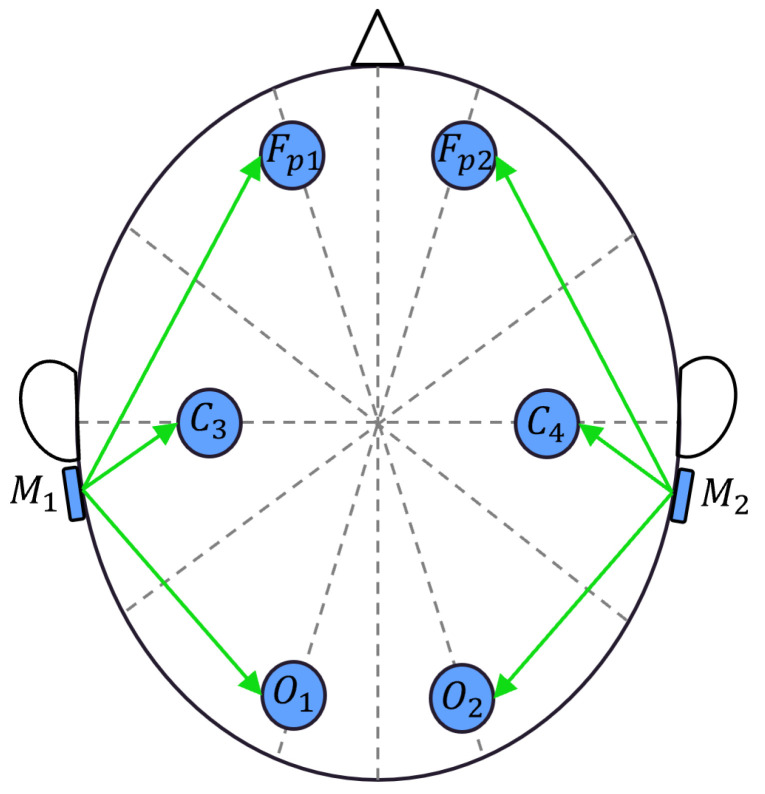
Placement of six-channel EEG sensor electrodes.

The EEG headband captures six EEG signals from the electrodes placed on the scalp according to the 10/20 standard [27]. The selected electrode positions include Fp1, Fp2, C3, C4, O1, and O2. The two mastoids, M1 and M2, serve as reference points for the left electrodes (Fp1, C3, O1) and the right electrodes (Fp2, C4, O2), respectively. The sensor measures the differential voltages between each electrode and the reference points, as shown in Figure 3. The signals are then conditioned by the sensor to convert low-level differential voltages with a full scale of ±1.5 mV to high-level single-ended voltages, with a full scale of 3.3 V. The conditioned signals are acquired by a 12-bit analog-to-digital converter, and through oversampling techniques, the data achieve a resolution of 14 bits, corresponding to 180 nV. The digitized signals are then sent to a Wi-Fi module via UART and transmitted wirelessly to a laptop via an access point. The system exhibits linearity in the order of 6 μV, operates at a sampling rate of 200 Sa/s, and has a bandwidth in the range of [0.8,44] Hz.

#### 2.1.2. SPR Sensors

The SPR sensors capture the differential voltages between the palm and the back of each hand, reflecting the electrical stimuli received by sweat glands from the autonomic nervous system. This signal acquisition is commonly referred to as endosomatic electrodermal activity. Similar to the EEG headband, the SPR sensors condition the signals to convert low-level differential voltages with a full scale of ±10 mV into high-level single-ended voltages with a full scale of 3.3 V. A 12-bit analog-to-digital converter is used to achieve a resolution of 4.8 μV. The digitized signals are then sent to a Wi-Fi module via UART and transmitted wirelessly to a laptop via an access point. The sensor system demonstrates linearity in the order of 30 μV, operates at a sampling rate of 200 Sa/s, and has a bandwidth in the range of [0.08,8] Hz.

#### 2.1.3. ECG Sensor

For ECG measurements, four disposable Ag/AgCl electrodes are used to capture two derivations on the chest. The ECG sensor conditions the signals to convert low-level differential voltages with a full scale of ±5 mV into high-level single-ended voltages with a full scale of 3.3 V. Similar to the EEG and SPR sensors, a 12-bit analog-to-digital converter is employed to achieve a resolution of 2.4 μV. The digitized signals are then sent to a Wi-Fi module via UART and transmitted to a laptop. The ECG sensor acts as an access point to collect data from the EEG and SPR sensors and then transmits all of the data wirelessly to a laptop. The system exhibits linearity in the order of 5 μV, operates at a sampling rate of 200 Sa/s, and has a bandwidth in the range of [0.08,75] Hz.

### 2.2. Experimental Setup

**Figure 4:**
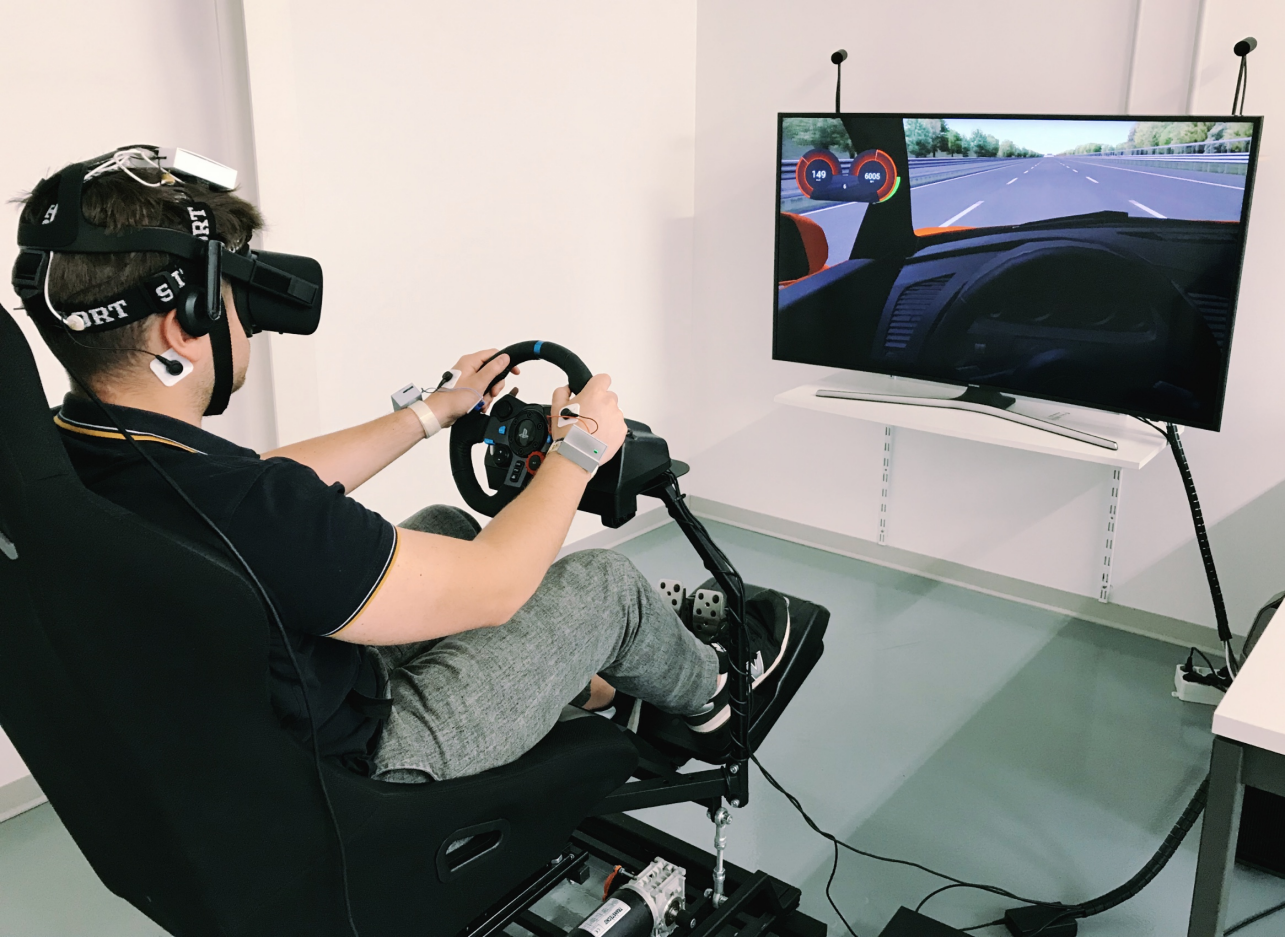
An individual participating in a manual driving scenario on our simulator. SPR, ECG, and EEG sensors were used to log the related physiological signals from the subject.

An experiment was carried out in the BioSensLab laboratory [28], involving a group of 19 participants comprising students and employees of the University of Udine. The age of the participants was in the [20,37] year range, with an average driving experience of approximately 7 years. All participants were in good health, free from any neurological or psychiatric disorders, and had normal or corrected-to-normal vision. They were instructed to abstain from consuming alcohol, caffeine, and nicotine for a minimum of 8 hours before the experiment. Prior to the experiment, participants were provided with detailed information about the study and were required to read and sign an informed consent form to record their physiological signals. For the experiment, sensors were placed on the head of each participant for EEG recordings, on both hands to capture SPR signals, and on the chest to record ECG signals. Detailed illustrations of sensor placements can be found in Figure 2 and Figure 4. The driving simulation setup used in the experiment comprised a three-axis moving platform (DOF Reality Professional P3), a curved screen, a Virtual Reality (VR) headset (Oculus Rift), a racing seat, and a force-feedback steering wheel with pedals and a gearbox (Logitech G29). This combination of equipment provided a realistic driving experience for the participants.

**Figure 5:**
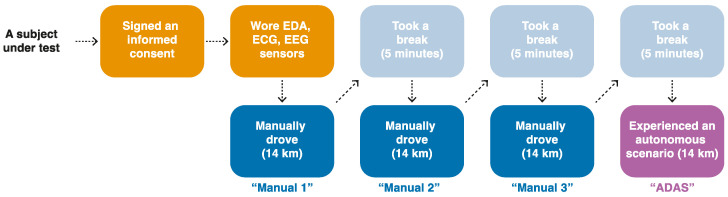
The sequence of experimental sessions.

At the beginning of the experiment, participants were instructed to wear the VR headset and engage in a total of four simulation sessions, three of which involved manual driving, and one was an autonomous driving simulation. These sessions simulated real-world driving conditions on a three-lane highway. Participants were asked to obey speed limits, avoid accidents, and navigate through road works. Figure 5 provides a visual representation of the different phases experienced by each participant during the experiment.

**Figure 6:**
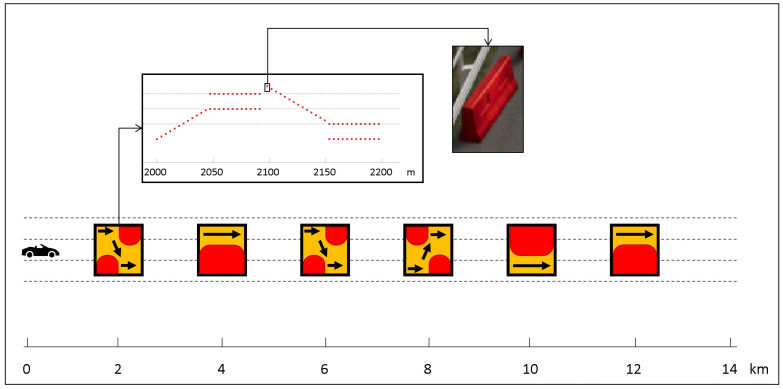
Manual1 highway track including six Jersey barriers. The first jersey barrier, which indicates a left to right pass, is shown in detail.

All participants started with manual driving on a 14 km highway route that included six obstacles positioned along the way. These obstacles were placed in six sections of the highway, each characterized by road works indicated by Jersey barriers. Barriers were randomly selected from four different types: right-to-left pass, left-to-right pass, right pass, and left pass. Each obstacle spanned a length of 200 m, with distances of 2 km between them. The first obstacle was located 2 km from the starting point of the course. This phase, referred to as “Manual1”, was completed by the participants in approximately 7 min, with an average driving speed of around 120 km/h. The Manual1 track is illustrated in Figure 6.

Following the completion of the Manual1 phase, participants were directed to take a 5 min rest period, during which they could walk away from the simulator. The subsequent phase, known as “Manual2”, replicated the conditions of Manual1, including the track length, the number of obstacles, and the instructions given to the participants regarding their driving. However, the order of the obstacles was randomly altered. Once Manual2 had been concluded, participants once again had a 5 min rest period. Following this, the participants started the third driving phase, referred to as “Manual3”, which required them to manually drive along the same highway, encountering six obstacles as before.

Upon completion of Manual3, the participants had another 5 min rest period. This pause led to the final phase, denoted as “ADAS”, which involved autonomous driving. During this phase, participants remained seated in the simulator while the vehicle journeyed without their active participation in the driving process. The first part of the track layout remained the same as before, with the six obstacles positioned at identical locations. Therefore, the first seven minutes of this phase replicated the previous manual driving scenarios, covering a distance of 14 km. Subsequently, an additional 13 min (27 km) of autonomous driving was conducted on the same highway but without any obstacles. This extended duration aimed to gather additional data for the ADAS scenario, as further explained in the subsequent section.

### 2.3. Synchronization of the Sensors

In this study, all developed sensors were synchronized within the same BSN in real time during signal acquisition. In this setup, the ECG sensor served as a gateway for transmitting the data from all sensors to the computer using the UDP protocol. The ECG sensor acquires two channels of ECG signals at a frequency of 200 Hz, while the SPR sensors acquire endosomatic EDA signals at the same frequency. Additionally, the ECG sensor has the task of receiving eight UDP packets from each SPR sensor every 40 ms, aligning them with the ECG signals, and creating a new UDP packet that includes eight samples from each SPR sensor and eight samples from each ECG channel. These packets are then transmitted to the computer every 40 ms. A complete description of ECG and SPR synchronization can be found in our previous article [26]. Similarly, the EEG sensor acquires EEG signals from six channels at a frequency of 200 Hz. The sensor generates UDP packets containing eight acquired samples from all six channels every 40 ms, which are then transmitted to the computer through the ECG sensor.

**Figure 7:**
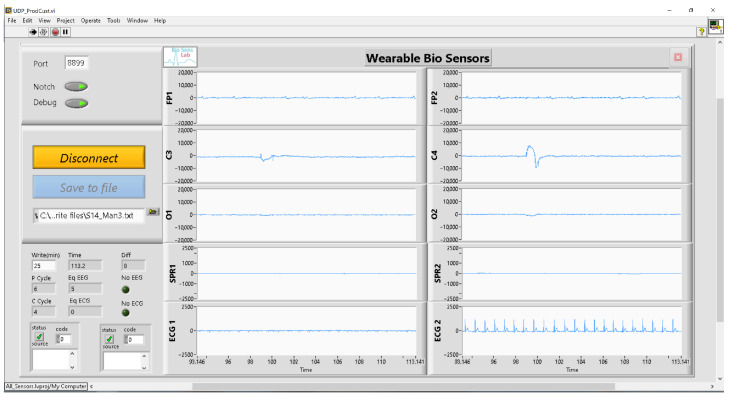
Developed GUI front panel.

To facilitate data collection, synchronization, visualization, and storage from each sensor, a custom GUI was developed in the LabView environment [29]. The GUI front panel, illustrated in Figure 7, presents real-time plots of signals acquired from all sensors. It includes a “connect/disconnect” button for initiating and halting signal acquisition. The acquired signals can be conveniently saved to a designated folder using the “save to file” button. Additionally, the GUI offers the option of applying a notch filter for eliminating unwanted power line noise. The GUI, running on the computer, receives the UDP packets from the ECG and EEG sensors, unpacks and aligns them, prepares the time base, and creates a dataset that includes samples from all sensors for each time instant. The dataset consists of the following columns in this order: time, Fp1, Fp2, C3, C4, O1, O2, SPR1, SPR2, ECG1, ECG2. In cases where a sensor stops or delays the transmission of data due to a shortage in the wireless connection or internal clock drift, the GUI handles these missing samples by inserting zeros into the dataset. Throughout the experiment, there were no instances of lost wireless connection, and only a few zeros were inserted into the dataset due to the sensor clock drifting. To ensure data integrity, these zero values were linearly interpolated between the preceding and succeeding samples.

Furthermore, data from the driving simulator software (Drivesim from VI-grade [30]) were manually synchronized with the dataset created by the GUI. To achieve this, at the beginning of each driving session, the “connect” button in the GUI was pressed simultaneously with the start button in the Drivesim software, which initiates the simulation and collects simulation data in a CSV file. The CSV file contains vehicle dynamics data, including lateral and longitudinal vehicle positions, sampled at a rate of 200 Sa/s. As the computer clock is more reliable compared to the sensors’ internal clocks, the length of the CSV file was used to determine the duration of the experiment. Consequently, the dataset generated by the GUI was adjusted to match this duration.This synchronization process allowed for accurate assignment of obstacles’ positions in the signals and facilitated the removal of corresponding portions of data in a synchronized manner, as detailed in the following section.

### 2.4. Signal Pre-Processing

During the experiment, biosignals were collected from all subjects using the designed biosensors described in Section 2.1. Ten signals were synchronously acquired from each subject with a consistent time base. These signals comprised six EEG signals, two SPR signals, and two ECG signals. During signal acquisition, to eliminate power line noise, the GUI’s notch filter (bandstop Butterworth filter) was applied with low and high cutoff frequencies of 46 Hz and 54 Hz, respectively, to the acquired signals. Before the classification stage, all recorded signals were pre-processed.

#### 2.4.1. EEG Signals

The six EEG signals acquired from the frontal region (Fp1, Fp2), the central region (C3, C4), and the occipital region (O1, O2) of the subjects’ scalps underwent a series of pre-processing steps to remove artifacts and enhance their overall quality. These signals, measured by surface electrodes, reflect the field potentials generated by collective neuronal activity primarily within the cerebral cortex situated beneath the skull. As a result, the EEG signals are attenuated by layers of skull and skin and are typically in the range of 10–100 μV in healthy adults. The relatively low amplitudes of the signals make them prone to several forms of artifacts. Specifically, two primary sources contribute to the occurrence of these artifacts. The first source includes additional physiological signals such as cardiac and muscle activities, eye movements, and blinks. The second source involves external factors such as AC power line noise, cable movements, and broken wire, which can further contribute to signal distortions [31].

**Figure 8:**
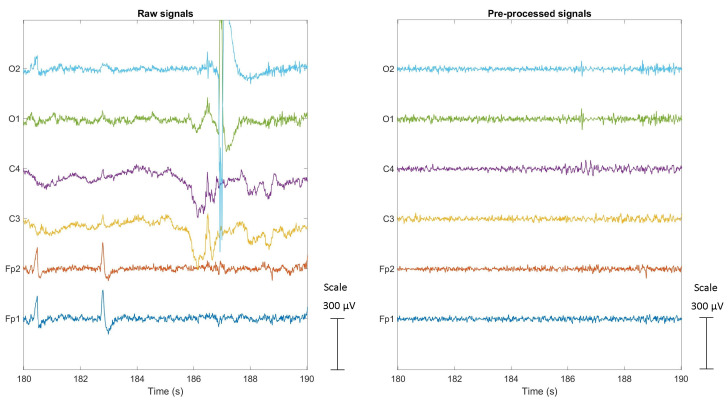
Raw and cleaned EEG signals during the Manual1 driving of a subject. Blue, red, yellow, purple, green, and cyan lines correspond to the Fp1, Fp2, C3, C4, O1, O2 signals, respectively.

Given the susceptibility of EEG signals to artifacts, several pre-processing steps were applied through a pipeline to the recorded signals using the freely available EEGlab Matlab toolbox [32]. The first step involved the visual inspection of all signals to identify segments that were heavily contaminated by artifacts. These segments were eliminated along with the corresponding portions of the ECG and SPR signals to maintain synchronicity among the signals. In the second step, the EEG signals went through high-pass and low-pass Finite Impulse Response (FIR) filters. The high-pass filter with a cutoff frequency of 4 Hz was applied to remove low-frequency drift and baseline fluctuations, while a low-pass filter with a cutoff frequency of 45 Hz was used to attenuate high-frequency noise and artifacts. To further improve the signal quality and remove the remaining artifacts, the Artifact Subspace Reconstruction (ASR) algorithm [33] was applied to all EEG signals. ASR utilizes statistical techniques and Principal Component Analysis (PCA) to identify and remove artifact components, resulting in a cleaner and more reliable signal. The outcome of the artifact removal pipeline is depicted in Figure 8. The figure shows a comparison between the raw EEG signals on the left and the cleaned signals on the right, both recorded during manual driving of a subject. The pipeline successfully eliminates artifacts, including baseline drift from the C3 and C4 signals, blinks and eye movements from the Fp1 and Fp2 signals, and electrode displacement artifacts from the O1 and O2 signals.

#### 2.4.2. SPR Signals

**Figure 9:**
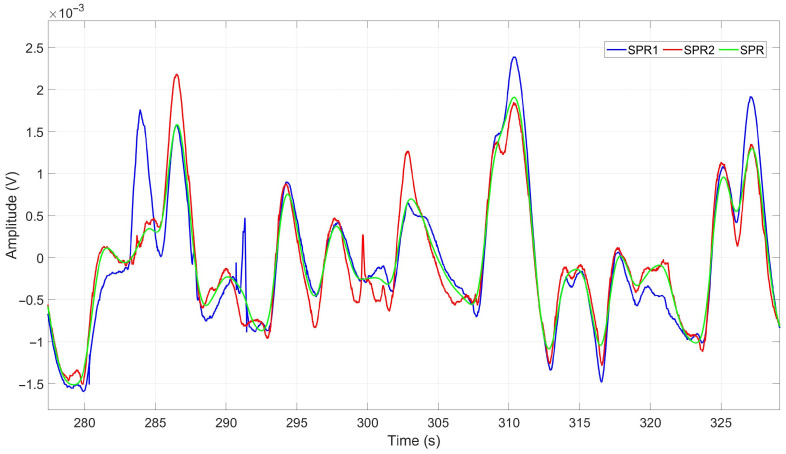
Raw and cleaned SPR signals during Manual1 driving of a subject. Blue (SPR1) and red (SPR2) are raw signals and green (SPR) is the cleaned signal output result of the motion artifact removal block.

The two SPR signals captured from each subject’s hands were input into a motion artifact removal block, designed to eliminate potential artifacts that may have occurred during driving. The algorithm employed in this step is based on the calculation of the local energy of the two input signals, as described in [34]. The resulting output of this process is a single cleaned SPR signal. In other words, for each time instant, it captures the smoother behavior between the two signals, representing a signal with fewer artifacts. Figure 9 displays the SPR signals acquired from a subject during manual driving. The blue and red signals, denoted as SPR1 and SPR2, respectively, represent the raw signals obtained from the subject’s right and left hands. The green signal, labeled as SPR, represents the output signal after passing through the motion artifact removal block, effectively eliminating potential artifacts.

#### 2.4.3. ECG Signals

**Figure 10:**
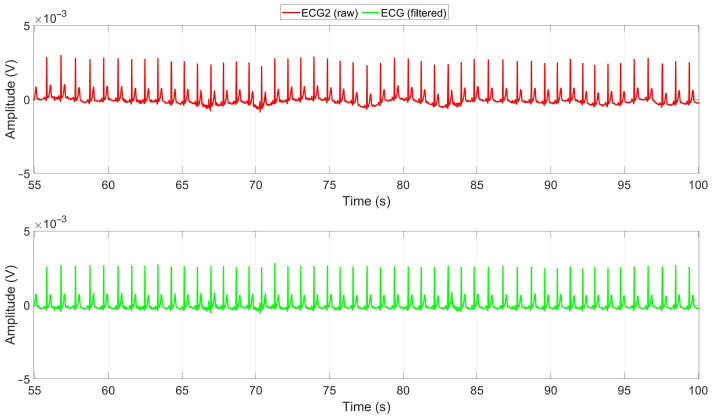
Raw and cleaned ECG signals during Manual1 driving of a subject. Red is the raw ECG2 signal, and green is the cleaned ECG signal after applying a high-pass filter with a cutoff frequency of 0.5 Hz.

The ECG signals obtained from the chest of each subject were evaluated, and the signal with the highest SNR was chosen. To improve the signal quality and eliminate the baseline drift and artifacts caused by respiration, a high-pass Infinite Impulse Response (IIR) filter with a cutoff frequency of 0.5 Hz was applied to the selected signal [35]. The effectiveness of this filtering process can be observed in Figure 10. The figure illustrates the raw and filtered ECG signals recorded during manual driving of a subject. The red signal represents the raw ECG signal, while the green signal corresponds to the cleaned signal obtained after applying the high-pass filter. The filter effectively removes baseline drift, enhancing the signal quality.

Subsequently, in order to effectively eliminate all motion artifacts from all signals, the signal portions corresponding to the obstacle-overcoming events were removed. This step was taken to account for the physical movements of the drivers and the artifacts that may have been generated as they encountered and maneuvered around the obstacles. To ensure precision, each obstacle was considered to span an additional 500 m (both before and after) beyond its actual duration of 200 m, and these extended portions were removed from the signal dataset.

#### 2.4.4. Signal Segmentation

Finally, the pre-processed signals were divided into non-overlapping time segments, each lasting 3 seconds. The length of optimal segment windows was determined experimentally, and a detailed discussion of our findings is presented in Section 3. Each segment consisted of a total of eight signals, comprising six cleaned EEG signals, a cleaned SPR signal, and a filtered ECG signal, all acquired with a sampling rate of 200 Hz. Thus, each segment contained 600 samples of the eight signals, extracted 600 samples after the previous one. The experiment was designed to induce high and low mental engagement in drivers through manual and autonomous driving tasks, respectively. This approach was chosen based on our previous research findings, which showed that manual driving elicits higher mental engagement compared to autonomous driving, and these differences can be detected through EEG, SPR, and ECG signals [36,37]. Accordingly, to represent high engagement, a label of “0” was assigned to all manual driving segments, while a label of “1” was assigned to all autonomous driving segments, representing low engagement.

### 2.5. Sensor Fusion Architectures

As previously detailed, signals from the EEG, SPR, and ECG sensors were collected and synchronized in real time during the experiment. Subsequently, the collected data were pre-processed and labeled according to the levels of high and low engagement of the drivers. Following the pre-processing stage, the data were used for classification in two different DL architectures by employing the BD-Deep4 model. These architectures are based on data-level fusion and feature-level fusion approaches and are described in the subsequent sections. We implemented all architectures using the Python programming language, taking advantage of the open-source TensorFlow and Keras libraries [38]. The entire data collection, pre-processing, and processing phases, including the training and testing of the DL models, were performed using a Dell XPS 15 9560 laptop with specifications including an Intel(R) Core(TM) i7-7700HQ CPU @ 2.80 GHz, 32 GB RAM, and an NVIDIA GPU GeForce GTX 1050.

#### 2.5.1. Data-Level Fusion

Biosignals exhibit complex patterns and forms that are dynamic and non-linear in nature. EEG signals, for instance, are characterized by low-amplitude and high-frequency brain waves, which are non-stationary over time [39]. As a result, extracting meaningful features from EEG signals presents a significant challenge. However, deep ConvNets have been recognized for their ability to effectively address this challenge for EEG, ECG, and EDA signals [40,41,42,43,44,45,46]. ConvNets excel at extracting local, low-level features from the raw input data in their initial layers and progressively capturing more global and high-level features as the network deepens. This ability to extract informative features from raw data makes ConvNets suitable for real-time online applications.

In this study, we adopted a widely used four-layer ConvNet model called Braindecode Deep4 ConvNet or BD-Deep4, as introduced in [24]. This multipurpose model has been applied in various studies with different applications, such as [40,47,48,49]. The model was designed to take a 2D array as the input, where the array’s width corresponds to the number of samples, and its height represents the number of electrodes. The model comprises four convolutional and max-pooling blocks. The first block includes two convolution layers, the first operating over time and the second operating over spatial channels, followed by a max-pooling layer. The subsequent three blocks consist of one convolution layer and one max-pooling layer each. The final block incorporates a dense layer with softmax activation for the purpose of two-class classification. Further details on the BD-Deep4 architecture can be found in the original paper [24].

**Figure 11:**
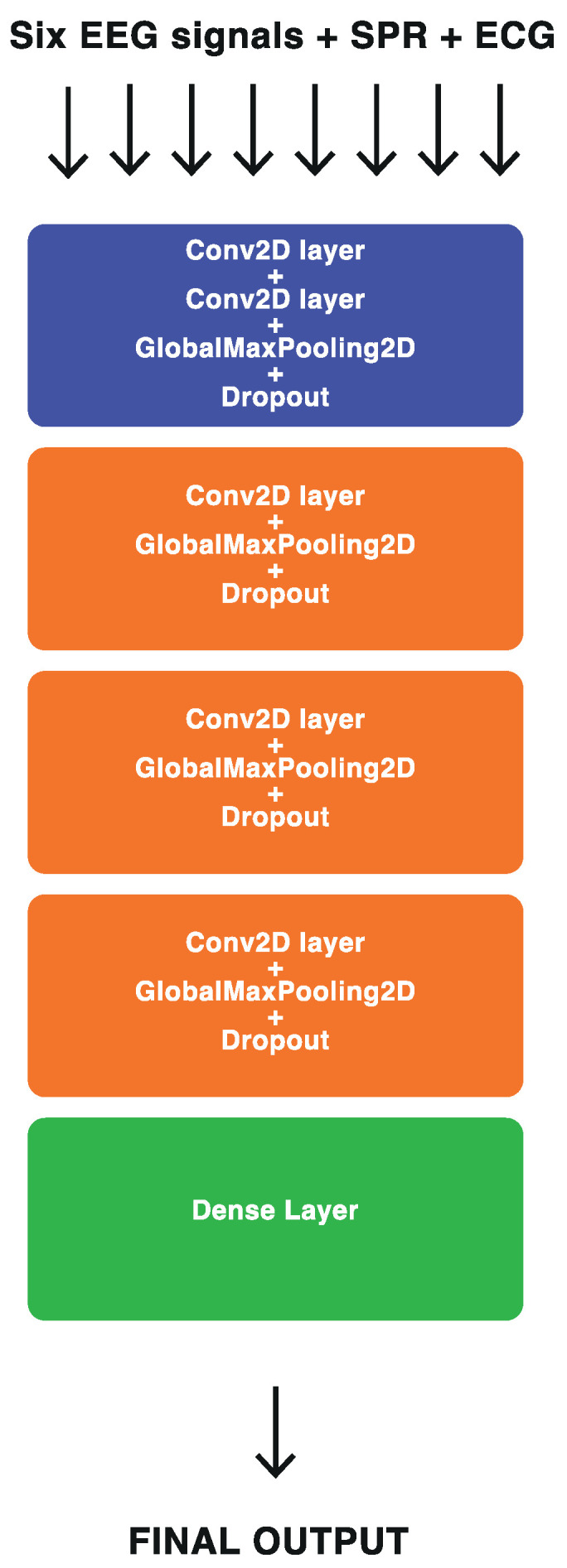
Block representation of the data-level fusion using BD-Deep4 architecture introduced in [24].

The BD-Deep4 model was implemented using different input configurations to assess the benefits of sensor fusion at the data level. As previously mentioned, all acquired signals were sampled at a rate of 200 Sa/s and segmented into 3 s windows. In the first configuration, the model received only the six channels of EEG signals as the input, resulting in a 2D input array, 600 × 6 in size. In the second configuration, the synchronized EEG and SPR signals were combined, creating a 2D input array, 600 × 7 in size. The third configuration incorporated the synchronized ECG signal with the EEG signals, resulting in a 2D input array of shape 600 × 7. Finally, the fourth configuration involved the combination of all synchronized signals (six channels of EEG, one SPR, and one ECG), forming an input array 600 × 8 in size. The last configuration is illustrated in Figure 11.

The model parameters were carefully selected based on the original article [24] as well as its implementation in [40]. To ensure optimal performance, the hyperparameters were fine-tuned through iterative experimentation. A summary of the model parameters can be found in Table 1. For the stochastic gradient-based optimizer, Adam [50] was selected as the preferred choice. To avoid overfitting, a regularization parameter (L2 penalty) of 1.3 was introduced for the dense layer. The categorical cross-entropy loss function was utilized to measure the model’s performance, and the training process was limited to a maximum of 200 epochs. Early stopping was also enabled, with training stopping after 50 epochs if no further improvement was observed. A batch size of 128 was employed during the training process. The evaluation methods used in this study, along with the comparison of the results obtained from different configurations, are detailed in Section 3.

#### 2.5.2. Feature-Level Fusion

**Figure 12:**
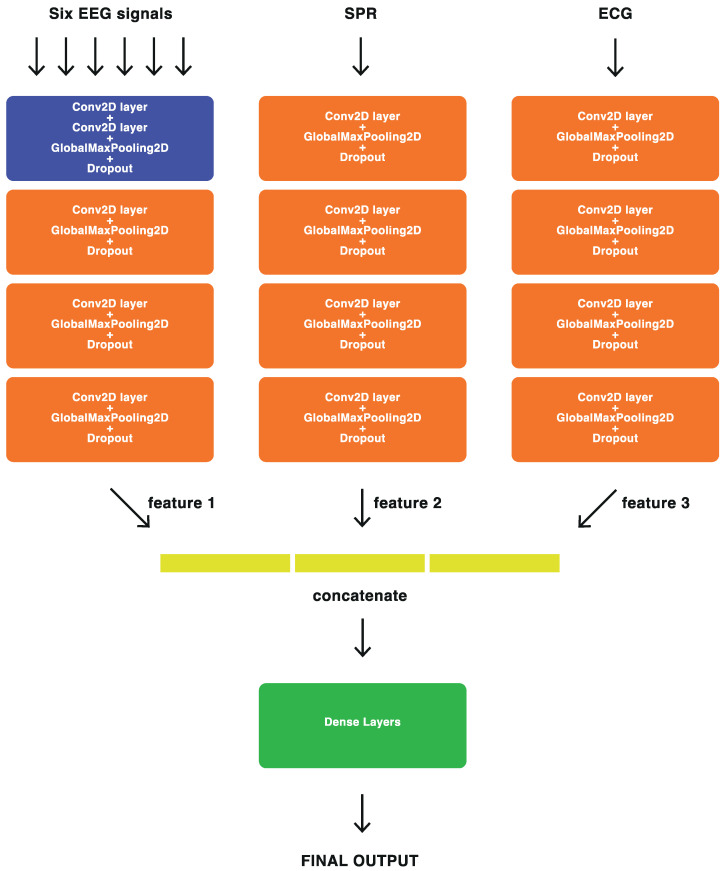
Block representation of the feature-level fusion ConvNet-Fus architecture.

For feature-level fusion, we employed our proposed DL architecture, where the most significant features extracted by the BD-Deep4 model [24] from each signal modality (EEG, ECG, and SPR) were combined to create a new fusion architecture, named ConvNet-Fus. This fusion architecture consists of three separate BD-Deep4 branches: one for EEG signals, one for SPR signals, and one for ECG signals. These three BD-Deep4 branches are then concatenated to combine the features extracted by each individual model. The BD-Deep4 branch responsible for extracting EEG features comprises four convolutional and max-pooling blocks. The first block includes two convolution layers followed by a max-pooling layer. The subsequent three blocks consist of one convolution layer and one max-pooling layer each. The two BD-Deep4 branches that extract SPR and ECG features have the same four blocks, with the distinction that the first block has only one convolution over time. This is because SPR and ECG signals have single channels and do not require convolution over space. The final block of ConvNet-Fus incorporates a concatenation layer to combine features obtained from each individual BD-Deep4 branch. This is followed by two dense layers, with the last layer utilizing softmax activation for the two-class classification. To assess the advantages of feature-level sensor fusion, the architecture of the ConvNet-Fus was modified to extract the features from EEG signals only, then those from EEG and SPR signals, then those from EEG and ECG signals, and finally those from EEG, SPR, and ECG signals. The detailed architecture of ConvNet-Fus is illustrated in Figure 12.

Similar to the BD-Deep4 model used for data-level fusion, the model parameters for ConvNet-Fus were selected based on the original article [24] and its implementation in [40] and fine-tuned through experimental iterations. The model parameters are summarized in Table 2. For the ConvNet-Fus model, Adam was again chosen as the preferred optimizer. To address overfitting, a regularization parameter (L2 penalty) of 0.7 was introduced in the dense layers. The categorical cross-entropy loss function, commonly used for classification tasks, was employed to measure the performance of the ConvNet-Fus model. During the training process, the maximum number of epochs was set to 50. Early stopping was implemented during training, which allowed the process to stop after 20 epochs if no significant improvement was observed. A batch size of 128 was employed during the training process. A detailed comparison of the results obtained from different configurations is provided in Section 3.

## 3. Experimental Results

In this section, the results obtained by applying the presented models to the physiological signals recorded from the test subjects are presented. The results include models for both the data-level fusion and feature-level fusion approaches. As described in Section 2.4, after the pre-processing steps, the synchronized data were segmented into 3 s windows, which were then labeled as “0” for high engagement intervals or “1” for low engagement intervals. The experiment involved a total of 19 subjects who participated in both manual and autonomous driving, resulting in a dataset comprising 7366 intervals in total.

### 3.1. Evaluation Method

The performance of the presented models was assessed using LOSO cross-validation, a robust and unbiased [51] technique that ensures subject independence in evaluation results. In this approach, the models were trained and tested iteratively for all subjects, with the training phase utilizing all intervals from all subjects, except the one being tested. By repeating this process for all subjects, the evaluation accounted for different subjects, making the results reliable and free from bias. The final performance metrics were computed by averaging the results obtained from each iteration. To mitigate biased predictions and enhance generalization, the training set was balanced before the training phase. This balancing process aimed to address any significant disparities in the number of samples across the two classes. On the other hand, the test set remained imbalanced, including all intervals extracted from subjects during driving activities.

To compare the results of the presented models, various performance metrics were calculated by averaging the results obtained for all subjects. The metrics considered included the accuracy, sensitivity, specificity, Balanced Accuracy (BA), and Geometric Mean (GM), which were computed as follows:

(1) $$Accuracy(\%)=\frac{TP+TN}{TP+TN+FP+FN}\cdot 100$$

(2) $$Sensitivity(\%)=\frac{TP}{TP+FN}\cdot 100$$

(3) $$Specificity(\%)=\frac{TN}{FP+TN}\cdot 100$$

(4) $$BA(\%)=\frac{1}{2}\left(\frac{TP}{TP+FN}+\frac{TN}{FP+TN}\right)\cdot 100$$

(5) $$GM(\%)=\sqrt{\frac{TP}{TP+FN}\cdot \frac{TN}{FP+TN}}\cdot 100$$

These parameters were derived from the numbers of True Positives (TP), False Positives (FP), True Negatives (TN), and False Negatives (FN). Sensitivity and specificity metrics measure the positive class classification and negative class classification performances, respectively. The significance of the BA [52] and GM [53] metrics lies in their independence from the distribution of classes. This characteristic makes them particularly valuable for the LOSO case, where the test set is imbalanced due to the varying driving durations in manual and autonomous scenarios.

### 3.2. Evaluation Results

Table 3 reports the final performance levels of the presented models, applying the data-level and feature-level fusion approaches. The performance parameters are presented as (MEAN ± STD) values, representing the mean values of the results obtained for all subjects, along with the corresponding standard deviations.

Considering the results for data-level fusion, it is evident that the performance indicators show similar values across different sensor combinations. Notably, when the model was solely fed with the six-channel EEG, the lowest performance was observed. However, a slight performance increase was observed with the addition of the SPR signal, resulting in an accuracy improvement from 69.8% to 70.2%. Furthermore, incorporating the ECG signal alongside the six-channel EEG signals led to a notable increase in accuracy of approximately 4%, raising it from 69.8% to 74.0%. However, the combination of all signals (six-channel EEG, SPR, and ECG) yielded a satisfactory accuracy level of 73.0%, slightly lower than the EEG and ECG combination. These results suggest that the addition of SPR signals to EEG signals at the data level has a limited impact on the final result, potentially due to the slower nature of the SPR signals compared to the EEG and ECG signals. Conversely, the combination of EEG and ECG signals in the data-level approach demonstrates the potential to enhance the performance of the BD-Deep4 classifier, exceeding the performance achieved by utilizing all sensor signals. This is evident from the notable improvement not only in the accuracy but also in the sensitivity, specificity, BA, and GM.

Taking into account the results of the feature-level fusion, it is worth noting that using only EEG signals as the input for feature extraction resulted in the lowest performance, similar to the data-level fusion approach. However, the inclusion of SPR features in addition to the six-channel EEG features led to a notable improvement, increasing the accuracy from 64.8% to 69.4%. In this architecture, it appears that BD-Deep4 was able to extract more meaningful features from the SPR signals individually compared to the data-level fusion case, where the SPR signals were combined with the EEG signals in the input 2D array. Nevertheless, despite this improvement, the performance level remained relatively low. Interestingly, when the ECG features were combined with the six-channel EEG features, the results improved even further, achieving an accuracy level of 80.9%. Moreover, the concatenation of features from all sensors through the ConvNet-Fus model achieved the highest performance, reaching an accuracy level of 82.0%. These findings indicate that this architecture, which relies solely on the features extracted from the six-channel EEG signals, yields a relatively low level of performance. The addition of the SPR signal features improved the overall accuracy by more than 5%, but the results were still not satisfactory. On the other hand, combining the EEG features with the ECG features resulted in a significant improvement, increasing the accuracy by more than 16% and producing notable results. Finally, the combination of the features derived from all sensors through ConvNet-Fus yielded a result that was slightly higher, by one percent, than the combination of EEG and ECG features, which is also a noticeable improvement. Considering other metrics, it can be noticed that the combination of all sensor features showed the best values with the least variation, suggesting that this configuration has the highest performance potential.

When comparing the results of the data-level fusion with those of the feature-level fusion, several key observations can be made. Firstly, it is evident that using only EEG signals in both approaches yielded the lowest performance. Moreover, in the case of utilizing only EEG signals, the data-level fusion approach (which corresponds to the original BD-Deep4 model from [24]) outperformed the feature-level approach, which incorporates a single branch of the same BD-Deep4 model with an additional dense layer consisting of 500 neurons placed before the final dense layer. This finding suggests that the original architecture was already optimized specifically for EEG signals, and making even minor modifications had a detrimental impact on the model’s performance. However, significant improvements in the fusion performance were observed with the newly designed architecture, which incorporates individual BD-Deep4 branches for each sensor’s signals and introduces a dense layer with 500 neurons. These enhancements were particularly notable when SPR and ECG features were added to EEG features, either individually or in combination. The improvements can be attributed to multiple factors. Firstly, BD-Deep4 effectively extracts meaningful features from each sensor’s signals, and their fusion through a concatenation step enhances the robustness and precision of the results. Secondly, the added dense layer with 500 neurons plays a crucial role in enhancing the performance. This was evidenced by the significantly lower accuracy achieved when we tested the same feature-level architecture without the dense layer for the combination of EEG, SPR, and ECG features, yielding an accuracy level of only 73.0% ± 22.0. This indicates that the dense layer effectively combined the extracted features from the EEG, SPR, and ECG signals, potentially enabling the model to learn higher-level abstractions. The 500 neurons in the dense layer allowed the model to capture more complex features, contributing to improved generalization capabilities.

#### 3.2.1. Results Distribution

**Figure 13:**
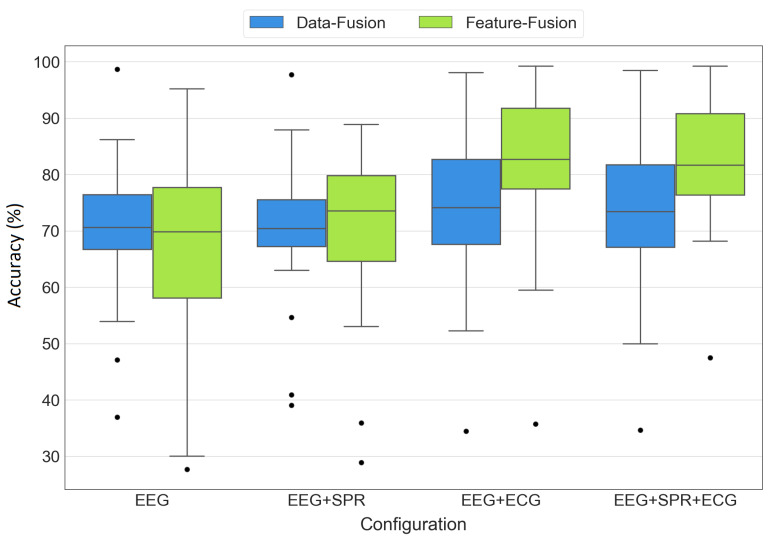
Boxplots of accuracy values obtained from the EEG, EEG+SPR, EEG+ECG, and EEG+SPR+ECG configurations for the data fusion (**blue**) and feature fusion (**green**) architectures.

To provide a comprehensive view of the statistical distribution of the results, Figure 13 displays the boxplots for all configurations of both data fusion and feature fusion approaches. While Table 3 provides the mean ± standard deviation of the results, the boxplots offer a more comprehensive representation of the distribution of the accuracy values across the 19 LOSO iterations. Each box in the plot represents the interquartile range, spanning from the first quartile (lower edge) to the third quartile (top edge), with a line inside the box representing the median of the results. The whiskers extend from the box to the minimum and maximum values, showcasing the range of variability in the accuracy values. Additionally, the black dots indicate any outliers present in each set of the results. The boxes are grouped by the tested configurations (EEG, EEG+SPR, EEG+ECG, EEG+SPR+ECG), with blue representing the data fusion architecture and green representing the feature fusion architecture. The proposed feature fusion architecture integrating EEG, SPR, and ECG signals demonstrates its superiority once again, which is evident from its high median, low variability, and the presence of only one outlier with a higher accuracy compared to the other outliers. However, the feature fusion architecture with EEG and ECG signals also produced favorable results, with a high median and only one outlier with a high accuracy as well. Nevertheless, this configuration exhibits a slightly broader variability range compared to the EEG+SPR+ECG configuration, indicating that the addition of SPR signals positively influenced the variability and increased the outlier’s value. On the other hand, when observing the remaining boxes, it became evident that both data fusion and feature fusion approaches utilizing only EEG signals or EEG+SPR signals did not perform as well as the EEG+ECG and EEG+SPR+ECG configurations. This finding reinforces the significant impact of integrating EEG and ECG signals in both data fusion and feature fusion approaches to obtain improved performance.

#### 3.2.2. Confusion Matrices

**Figure 14:**
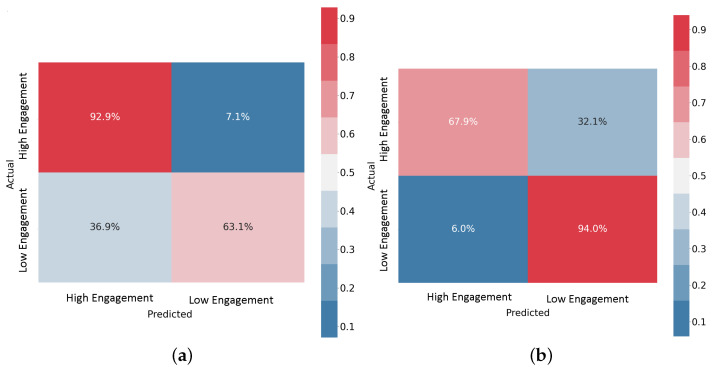
Confusion Matrices for the EEG+SPR+ECG configuration. (**a**) Data-level fusion model, (**b**) feature-level fusion model.

The Confusion Matrices (CMs) shown in Figure 14 illustrate the performances of the data-level fusion and feature-level fusion architectures with the EEG+SPR+ECG configuration. These CMs specifically depict the results for subject 11 during LOSO validation. The CM provides a visual representation of the predicted and actual segments (intervals) for both classes: high engagement and low engagement. Each row in the CM represents the actual segments of a specific class, while each column represents the predicted segments for a particular class. The main diagonal of the CM represents the correct predictions, whereas the off-diagonal elements represent the misclassifications. Values in the CM are presented as percentages of the total number of sample segments. It is worth noting that the data fusion model accurately predicted most of the segments in the high engagement class, and more than 60% of the segments in the low engagement class. Conversely, the feature fusion model accurately predicted most of the segments in the low engagement class and more than 65% of the segments in the high engagement class. When comparing the overall accuracy levels of the two models for subject 11, which is 75% for the data fusion model and 77.5% for the feature fusion model, it is evident that both models achieved acceptable results, with the feature fusion model performing slightly better. These findings highlight the effectiveness of both fusion approaches in integrating EEG, SPR, and ECG signals and their features to discern the mental engagement of drivers. The feature fusion model demonstrated superior performance in accurately classifying segments across both engagement classes.

#### 3.2.3. Accuracy Comparison among Subjects

**Figure 15:**
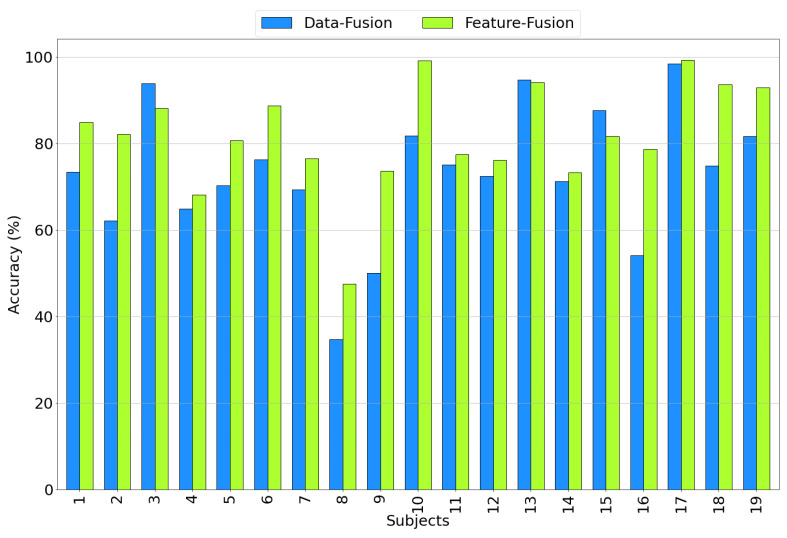
Accuracy obtained for each subject considering the EEG+SPR+ECG configuration for the data fusion (**blue**) and feature fusion (**green**) architectures.

Figure 15 illustrates the accuracy achieved for all subjects when considering both data and feature fusion approaches using the EEG+SPR+ECG configuration. A notable observation is that, in general, the accuracy for most subjects is higher in the feature fusion approach compared to the data fusion approach, with a few exceptions (subjects 3, 13, and 15). Subject 8 exhibits a relatively low accuracy of 34.7% in the case of data fusion, but this improved to 47.5% when employing feature-level fusion. Similarly, subject 9 and subject 16 show relatively low accuracy levels of 50% and 54.1%, respectively, in the data fusion approach, but these increased to 73.7% and 78.7%, respectively, when employing feature fusion. These results further emphasize the superior performance of the proposed feature fusion approach using the EEG+SPR+ECG configuration, as it consistently yields higher accuracy values across the majority of subjects.

### 3.3. Segmentation Window Length Comparison

**Figure 16:**
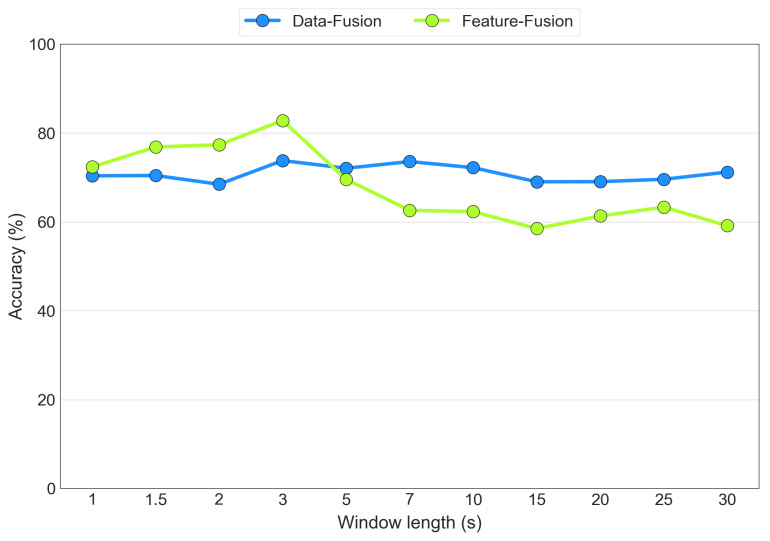
Accuracy obtained from the variation in the widow size, considering the EEG+SPR+ECG configuration for the data fusion (**blue**) and feature fusion (**green**) architectures.

As discussed previously, the optimal segmentation window length was determined experimentally through an iterative process for both the data fusion and feature fusion architectures. Figure 16 displays the average accuracy values as the segmentation window length varies for the EEG+SPR+ECG configuration for both the data fusion (blue line) and the feature fusion (green line) cases. The tested window lengths were determined on the basis of common segmentation lengths found in the literature. The figure illustrates that both models achieved their highest accuracy when using 3 s segmentation windows. However, the feature fusion model exhibited higher sensitivity to the window length, ranging from an 82% accuracy with 3 s windows to a 59% accuracy with 30 s windows. In contrast, the data fusion model demonstrated less sensitivity, with an accuracy level ranging from 73% with both 3 s and 7 s windows to 68% with 2 s windows. Consequently, we selected the 3 s window as the optimal choice for both the data fusion and feature fusion architectures.

### 3.4. Computational Cost

Table 4 presents a comparison of the trainable parameters, the number of epochs with early stopping patience values, and the computational time for one iteration of LOSO cross-validation across all data fusion and feature fusion architectures. Each LOSO iteration involves training the model on 18 subjects and testing it on the remaining subject. It is essential to note that the computational time can be subjective, depending on the computation system and its conditions. In our case, all computations were processed using an NVIDIA GeForce GTX 1050 GPU. The table reveals that the computational times were closely comparable among all models, as they were predominantly influenced by the memory access latency when loading datasets, which was consistent across all configurations. Notably, the feature fusion model using only EEG signals took the shortest time (164 s) but achieved the lowest accuracy of 64.8%. Conversely, the feature fusion model with EEG, SPR, and ECG signals required the longest time (325 s) but attained the highest accuracy. On the other hand, a notable disparity arose in terms of trainable parameters between the data fusion and feature fusion frameworks. Specifically, the data fusion model using only EEG signals had the fewest trainable parameters (149,477) while achieving an accuracy of 69.8%. In contrast, the model with highest performance, the feature fusion architecture integrating EEG, SPR, and ECG signals, contained the highest number of parameters (10,302,777) and achieved an accuracy level of 82%.

## 4. Discussion

In this study, we aimed to explore the benefits of sensor fusion by synchronizing multiple developed biosensors on the same BSN and implementing DL algorithms with multiple sensor fusion approaches. In previous works, we assessed the emotional states of drivers using machine learning approaches with either SPR signals only [54,55] or a combination of SPR and ECG signals [56,57,58]. In this work, we focused on evaluating the level of drivers’ mental engagement during manual and autonomous driving scenarios by employing advanced DL models that integrate EEG, SPR, and ECG signals.

### 4.1. Evaluation Method

In this research, we utilized the LOSO cross-validation technique, which involves repeating the training and testing processes for the subjects who participated in the experiment. While LOSO cross-validation is computationally expensive, it provides a robust estimation of a model’s performance on unseen subjects, enabling us to assess its generalizability. This approach prevents potential biases and overestimation of the model’s performance, which might occur with other subject-dependent methods [59,60].

### 4.2. Signal Modality Impact on Fusion

To assess the advantages of multimodal sensor fusion, we initially examined the performance of EEG signals alone, as they are the most complex signals. Subsequently, we evaluated the integration of additional signals (SPR and ECG) with EEG signals using both data fusion and feature fusion approaches. The results consistently showed that the addition of SPR and ECG signals led to performance improvements, with notable enhancements observed specifically through the integration of ECG signals with EEG signals in both fusion approaches. The relatively smaller improvement observed with the addition of SPR signals can be attributed to the deep ConvNet model (BD-Deep4) used in this study, which is more suitable for extracting meaningful features from complex signals such as EEG and ECG. It is possible that a different configuration for feature-level fusion, where fusion branch models are customized for the respective sensor signals, could yield better results. In our study, for the purpose of direct comparison with the data-level fusion architecture, we maintained the same BD-Deep4 model across all fusion branches. This approach facilitated a clear evaluation of the fusion techniques. Additionally, the relatively lower effectiveness of SPR integration may be attributed to the use of 3 s segmentation windows, which is a common practice for both EEG [61] and ECG [45] signals. Although the length of the windows was determined experimentally and optimized for both data fusion and feature fusion approaches, it is possible that EDA signals, characterized by their less dynamic nature, would benefit from longer segmentation windows. Nevertheless, despite these considerations, the integration of SPR signals with EEG signals still contributed to performance improvements, particularly in the feature fusion configuration. These findings reaffirm that integrating data from diverse biosensors not only enhances the system’s robustness but also improves the classification accuracy and overall performance.

### 4.3. Performance and Cost Trade-Off

As detailed in Section 3, the performance of the feature fusion approach outperformed that of the data fusion approach when integrating features from the EEG, SPR, and ECG sensors. This finding is consistent with the existing literature, which emphasizes the superiority of feature fusion for integrating heterogeneous sensors [9]. However, it is important to note that the feature fusion approach entails more complex algorithms and a greater number of trainable parameters, resulting in increased computational costs. Choosing the most suitable model involves finding a balance between the efficiency and computational cost, depending on the specific application. If computational cost is a primary concern, the data fusion model with EEG and ECG signals, offering an accuracy of 74%, provides a reasonable trade-off. Conversely, if efficiency takes precedence over the computational cost, the feature fusion model with the EEG, SPR, and ECG configuration, achieving an 82% accuracy, would be the preferred choice.

### 4.4. Intra-individual Variability

By observing the accuracy values achieved for subjects in the EEG+SPR+ECG configuration, considering both data fusion and feature fusion approaches, it became evident that several subjects obtained relatively low accuracy scores. This discrepancy can be attributed to the inherent nature of the intra- and inter-individual variability of physiological signals. Numerous factors can influence the physiological signals within a subject, highlighting the need for a larger and more diverse dataset to encompass the wide range of biological signal variations. In our study, we recruited 19 volunteers to participate in the experiment. However, it is important to acknowledge that a larger number of subjects would further enhance the accuracy and increase the generalization capabilities of the models.

## 5. Conclusions

In this study, we investigated the application of two different sensor fusion approaches using deep ConvNets and compared their respective results. The first approach involved the combination of sensor signals at the data level, while the second approach focused on feature-level fusion by employing our proposed architecture. The main objective was to assess the level of the driver’s mental engagement during simulated driving scenarios, including both manual driving and autonomous driving, where obstacles were placed along the road. The EEG, SPR, and ECG signals were recorded from the subjects using a real-time synchronization method facilitated by a custom-designed GUI. The collected data were subsequently pre-processed and labeled according to the corresponding levels of high and low mental engagement exhibited by the drivers. Various combinations of EEG, SPR, and ECG signals were tested to evaluate the impact of each type of signal fusion on the overall results. In the data-level fusion approach, the highest accuracy was achieved by combining the EEG and ECG signals, resulting in an accuracy of 74.0%. On the other hand, in the feature-level fusion approach, the combination of EEG, SPR, and ECG features through our proposed ConvNet-Fus architecture yielded the highest accuracy level of 82.0%. These findings demonstrate that feature-level fusion is the superior approach for integrating EEG, SPR, and ECG modalities, as it provides the best accuracy when detecting the mental engagement level of drivers. Furthermore, our proposed architecture exhibited promising performance in this regard. For future research, we intend to improve our architectures by incorporating specialized models designed specifically for each signal modality, aiming to achieve higher accuracy in the classification results. Moreover, we are focused on developing simplified models that are better suited for real-time applications. Additionally, we plan to design new experiments that align with publicly accessible datasets with an emphasis on driver state assessment. The future study will include a larger participant pool, thus enhancing the robustness and generalizability of our approach.

## Figures and Tables

**Table 1:** Implemented BD-Deep4 model parameters for the data-level fusion approach.

Layer	Filters/Nodes	Kernel Size	Pool Size, Stride
Conv. layer 2D	25	(1, 5)	
Conv. layer 2D	25	(N${}_{\mathrm{Channels}}$, 1)	
Batch normalization			
Activation (elu)			
Max-pooling 2D			(1, 2)
Dropout (0.38)			
— — — — — —			
Conv. layer 2D	50	(1, 5)	
Batch normalization			
Activation (elu)			
Max-pooling 2D			(1, 2)
Dropout (0.38)			
— — — — — —			
Conv. layer 2D	100	(1, 5)	
Batch normalization			
Activation (elu)			
Max-pooling 2D			(1, 2)
Dropout (0.38)			
— — — — — —			
Conv. layer 2D	200	(1, 5)	
Batch normalization			
Activation (elu)			
Max-pooling 2D			(1, 2)
Dropout (0.38)			
Flatten			
— — — — — — — — — —	— — — — — —	— — — — — —	— — — — — —
Dense (softmax)	2		

**Table 2:** Implemented ConvNet-Fus model parameters for feature-level fusion.

Branch	Layer	Filters/Nodes	Kernel Size	Pool Size, Stride
	Conv. layer 2D	25	(1, 5)	
	Conv. layer 2D ${}^{a}$	25	(N${}_{Channels}$, 1)	
	Batch normalization			
	Activation (elu)			
	Max-pooling 2D			(1, 2)
	Dropout (0.5)			
	— — — — — —			
	Conv. layer 2D	50	(1, 5)	
	Batch normalization			
	Activation (elu)			
	Max-pooling 2D			(1, 2)
	Dropout (0.5)			
	— — — — — —			
	Conv. layer 2D	100	(1, 5)	
EEG, SPR, ECG	Batch normalization			
	Activation (elu)			
	Max-pooling 2D			(1, 2)
	Dropout (0.5)			
	— — — — — —			
	Conv. layer 2D	200	(1, 5)	
	Batch normalization			
	Activation (elu)			
	Max-pooling 2D			(1, 2)
	Dropout (0.5)			
	Flatten			
— — — — —	— — — — — — — —	— — — — —	— — — — —	— — — — —
	Concatenate			
	Dense (relu)	500		
	Dense (softmax)	2		

${}^{a}$ For EEG branch only.

**Table 3:** Performance comparison of the data-level fusion and the feature-level fusion approaches.

Data-Level Fusion (Mean ± Std)					
**Fused Signals**	**Accuracy (%)**	**Sensitivity (%)**	**Specificity (%)**	**BA (%)**	**GM (%)**
EEG	69.8 ± 13.9	76.4 ± 31.3	57.0 ± 32.9	66.7 ± 14.0	56.4 ± 23.1
EEG+SPR	70.2 ± 14.1	76.8 ± 29.4	58.8 ± 33.7	67.8 ± 14.4	58.7 ± 22.1
EEG+ECG	74.0 ± 15.4	76.9 ± 29.0	69.7 ± 30.7	73.3 ± 13.5	65.8 ± 22.5
EEG+SPR+ECG	73.0 ± 15.4	76.3 ± 33.6	65.0 ± 33.1	70.6 ± 14.8	59.6 ± 27.1
**Feature-Level Fusion (Mean ± Std)**					
**Fused Features**	**Accuracy (%)**	**Sensitivity (%)**	**Specificity (%)**	**BA (%)**	**GM (%)**
EEG	64.8 ± 18.6	78.2 ± 35.1	42.9 ± 34.4	60.6 ± 14.8	43.0 ± 27.2
EEG+SPR	69.4 ± 15.5	87.3 ± 23.5	41.1 ± 32.7	64.2 ± 13.9	48.8 ± 28.1
EEG+ECG	80.9 ± 15.1	81.6 ± 29.1	76.4 ± 28.7	79.0 ± 16.6	71.7 ± 28.5
EEG+SPR+ECG	82.0 ± 12.0	82.4 ± 27.1	77.6 ± 23.6	80.0 ± 13.4	74.8 ± 22.8

**Table 4:** Comparison of the trainable parameters and computational time for data fusion and feature fusion architectures.

		EEG	EEG+SPR	EEG+ECG	EEG+SPR+ECG
Data Fusion	Parameters	149,477	150,102	150,102	150,727
	Epochs (ES ${}^{a}$)	200 (50)	200 (50)	200 (50)	200 (50)
	Time (s)	250	247	313	272
Feature Fusion	Parameters	3,437,777	6,870,277	6,870,277	10,302,777
	Epochs (ES ${}^{a}$)	50 (20)	50 (20)	50 (20)	50 (20)
	Time (s)	164	242	227	325

${}^{a}$ Early stopping patience value.

## Data Availability

The data presented in this study are available on request from the BioSensLab website https://www.biosenslab.it/resources.

## Abbreviations

The following abbreviations are used in this manuscript:

SPR	Skin Potential Response
EDA	Electrodermal Activity
EEG	Electroencephalogram
ECG	Electrocardiogram
DL	Deep Learning
ML	Machine Learning
ConvNet	Convolutional Neural Network
